# The Medical Defence Union and the London and Counties Medical Protection Society (Limited)

**Published:** 1893-12-09

**Authors:** Leslie Phillips, A. G. Bateman

**Affiliations:** Secretary to the Medical Defence Union; Secretary to the Medical Defence Union


### THE MEDICAL DEFENCE UNION AND THE LONDON AND COUNTIES MEDICAL PROTECTION SOCIETY (LIMITED).

Sir,—We observe in your last issue a letter from Dr. Hugh Woods purporting to be a criticism of some of the statements in the *résumé* of the amalgamation negotiations published by the Council of the Medical Defence Union. Dr. Hugh Woods distinctly implies that certain of these statements are untrue. We ask, therefore, for space to confirm the accuracy of that which was originally written.

The first allegation of Dr. Woods is that the conference was not terminated by the refusal of his society to accept the revision of the names of their representatives by the Council of the Defence Union. Owing to Dr. Woods having attended only a small proportion of the meetings of the Amalgamation Committee, he is doubtless unaware of the fact that his colleague, Dr. Heron, informed the Committee that the Council of the London and Counties Society wou.d definitely refuse to entertain for a moment such a proposal, and that if it were persisted in, the negotiations were at an end. On the matter being referred to our Council the original proposal was again confirmed, and again rejected by the delegates of the London and Counties Society.

What Dr. Woods refers to as a counter-proposal was really only an afterthought of Dr. Heron's, and had no meaning in that stage of the negotiations, since it had already been agreed to by the Joint Committee (see Par. No, 1 in the *résumé, et seq.*) to leave the Executive of the Union as it stood, the addition of the nominees of the London and Counties Society being in the shape of an appointment of Vice-Presidents to existing Council of the Union.

Dr. Woods' second allegation is so worded as to convey the impression to his readers that in the negotiations the winding up of the London and Counties Society was not contemplated. This serious and doubtless unintentional misrepresentation of what passed at the Joint Committee is probably due to the fact that he was again not present at the meeting at which this course was agreed upon by his colleagues, and at which also the solicitors representing the two societies assisted.

Dr. Woods' third allegation is that the statement of the Council of the Defence Union, to the effect that the numerical strength of the London and Counties Society is between 600 and 700 is untrue; further, that it is in reality between 800 and 900. In answer to this allegation, we have only to say that the estimate quoted by the Council of the Defence Union, namely, 600 or 700, was that furnished to the Joint Committee, and agreed to by those representatives of the London and Counties Society who happened to be present at that meeting, namely, Dr. Heron and Mr. Bruce Clark.

We note in passing that Dr. Woods imputes inaccuracy to the statement of the numerical strength of the Defence Union. For answer, we refer him to the register of members, which he ought in honour to have examined before putting forward so grave a charge.

Dr. Woods' fourth allegation is to the effect that our Union is disposed to act simply as a defender of its individual members. Of course, this statement is wholly untrue, as everyone who reads the report of the Defence Union is well aware.

Dr. Woods' fifth allegation is to the effect that the Executive of the Defence Union is an oligarchy, but that of his society is a free government. To this we only desire to reply that the Executive of the Defence Union is annually elected, whereas that of the London and Counties Society is at present elected for *three years* without any power on the part of the members to depose their rulers; and further that the Defence Union Council meetings are held fortnightly on fixed dates, and are attended by an average of twenty members drawn from all parts of the country, such frequent meetings absolutely ensuring that all matters are dealt with by the Council itself, and not by one or two members of its executive.

Dr. Wood's sixth allegation, to the effect that the medical officials are paid, is worded so vaguely, that it is difficult to comprehend what he intends should be understood by it. No medical officer of the Defence Union is paid any salary at all. At the general meeting in 1891, in consideration of the excessive clerical work which had been performed by the secretaries during the last two years, and amounting to some 12,000 letters, besides reports, &c., it was decided that an honorarium of fifty guineas be given to each secretary, and in 1892, when in that one year the letters alone exceeded 10,000, fifty guineas were paid to Dr. Bateman, and one hundred guineas to Dr. Leslie Phillips by the Union. As these are the only payments which have been made at all, the amount contributed by each member is rather less than three pence per annum. The work of the Union is now so very onerous as to render it in the view of the Council unjustifiable to demand gratuitous service involving heavy responsibility for objects which are of public and not private interest, the Council of the Defence Union being of opinion that the principle of remuneration which obtains in the General Medical Council should be applied to the office of secretaries to the Union.—Yours faithfully,

Leslie Phillips, M.D.
A. G. Bateman, M.D.
Secretaries to the Medical Defence Union.

